# Iodine in Cutaneous Diseases

**Published:** 1831

**Authors:** 


### XXXVII.

### IODINE IN CUTANEOUS DISEASES.

DR. JEFFRAY, of Liverpool, has stated in the Lancet, that he has administered the tincture of iodine in various cases of psoriasis, and the different varieties of herpes, "with almost never failing success," as well as in lepra vulgaris. In the latter case the diluted unguent. hydrarg. nitrat. was also employed. We observe that Dr. Jeffray begins with ten drops twice a day, increasing the dose to thirty drops night and morning.

---

